# Novel Protein Adjuvant Activating Innate IFN-γ and IL-18 Expression and Inducing Rejection of Implanted Colorectal Cancer Following Immunotherapy Using This Adjuvant in Mice

**DOI:** 10.18103/mra.v13i6.6615

**Published:** 2025-06-30

**Authors:** Rajesh Mani, B. Mark Evers, Yasuhiro Suzuki

**Affiliations:** 1 Department of Microbiology, Immunology and Molecular Genetics, University of Kentucky College of Medicine, Lexington, KY 40536, USA.; 2 Department of Surgery, University of Kentucky College of Medicine, Lexington, KY 40536, USA.; 3 Markey Cancer Center, University of Kentucky College of Medicine, Lexington, KY 40536, USA.

**Keywords:** adjuvant, cancer immunotherapy, IFN-γ, IL-18, innate immunity, protective immunity, cancer rejection

## Abstract

Our recent study demonstrated that immunizations with nonreplicable MC38 colorectal cancer cells plus a novel recombinant protein adjuvant, the amino-terminus region of dense granule protein 6 (rGRA6Nt) of *Toxoplasma gondii* (a protozoan parasite), effectively activate the cancer cell-specific CD8^+^ T cytotoxic cells and inhibit the growth of implanted tumors of the identical cancer cells after its challenge implantation. In the present study, we first examined whether rGRA6Nt activates mRNA expression for IFN-γ, IL-12, IL-15, and IL-18, which are known to assist an activation of the CD8^+^ T cells, in innate immune cells. Following an intraperitoneal injection of rGRA6Nt (40 μg) into SCID mice deficient in both T and B cells, markedly increased levels of mRNA for only IFN-γ and IL-18 were detected in their peritoneal exudate innate immune cells. When C57BL/6 mice were immunized with nonreplicable MC38 colorectal cancer cells plus rGRA6Nt adjuvant twice and challenged with replication-capable cancer cells of the identical colorectal cancer, more than one fifth (22.2%, 6/27) of the immunized mice rejected the growth of the implanted tumors, whereas none (0/27, *P*<0.05) of unimmunized control mice rejected the implanted tumors. These results indicate that rGRA6Nt protein adjuvant has a unique capability to selectively activate expression of IFN-γ and IL-18 in innate immune cells, and that immunizations with nonreplicable cancer cells in combination with this protein adjuvant can induce a protection to reject the growth of the identical tumor cells after its challenge implantation in a significant portion of the immunized mice.

## Introduction

There are two major recent advancements in cancer immunotherapy. One is chimeric antigen receptor (CAR) T cell therapy. CAR T cells express genetically modified antigen receptors composed of the antigen-specific binding component of a monoclonal antibody combined with T cell receptor signaling molecules required for T cell activation^1^. CAR T cells can specifically target the cancer cells expressing an antigen recognized by the CAR expressed by the those T cells. Although the immunotherapy using CD19-specific CAR T cells is quite effective for B cell lymphomas that commonly express CD19^1^, applying this method to treat other types of cancers is challenging because of variations of cancer antigens among different cancer types and individual patients.

Another key advancement in cancer immunotherapy is immune checkpoint blockage therapy. The immune system utilizes powerful proinflammatory responses to eliminate foreign materials such as pathogens and cancer cells. Because overly activated proinflammatory responses can cause severe tissue damages, the protective immune responses need to be finely tuned to prevent the overly stimulated responses. Immune checkpoint molecules such as PD-1 and CTLA-4 play important roles in the prevention of overly stimulated immune responses^[2-4]^. However, cancer cells often express these immune checkpoint molecules to avoid activation of the protective immune responses that attack the cancer cells. Treatment of cancer patients with the immune checkpoint inhibitors to block this immune suppression process has made a notable progress in cancer therapy^5–8^. However, since the effects of the immune checkpoint inhibitors are not specific to the anti-cancer immunity, this immunotherapy often causes unwanted overly stimulated immune responses to antigens unrelated to the cancer cells and causes potentially serious toxic side effects^9,10^. In addition, the immunotherapy with the immune checkpoint inhibitors have been showing only limited efficacy in treatment of some of more prevalent cancers including colorectal cancers (CRC)^5–8^.

CRC is the 3rd most prevalent cancer worldwide. Notably, 30–35% of the patients who receive curative surgical resection have recurrence of the cancer^11,12^. Therefore, there is an urgent need to develop a pathway to prevent recurrence of surgically resected CRC. Cancer-specific CD8^+^ cytotoxic T cells can kill cancer cells^[13]^, and the presence of tumor-infiltrating CD8^+^ T cells is an indicator of positive prognosis of cancer patients^14,15^. Thus, a valuable approach for preventing recurrence of surgically resected CRC could be the use of surgically resected own cancer cells for immunization to activate CD8^+^ cytotoxic T cells specific to their own cancer cells. However, cancer-specific antigens are often not strongly immunogenic. Therefore, the use of a potent adjuvant that is able to facilitate the activation of CD8^+^ cytotoxic T cells specifically to cancer cells of each of the patients is required for the immunotherapy using the cancer cells obtained from the patients.

We recently discovered that the amino-terminus region (amino acids 41–152) of dense granule protein 6 (GRA6Nt) of *Toxoplasma gondii*, an intracellular protozoan parasite, functions as a powerful adjuvant when used in immunization with nonreplicable MC38 CRC cells to activate cancer-specific CD8^+^ cytotoxic T cells, and that the immunization using GRA6Nt adjuvant markedly inhibits the growth of tumors of the identical CRC after its challenge implantation^16^. IFN-γ, IL-12, IL-15, and IL-18 have been shown to promote an activation of CD8^+^ T cells^17–20^. In the present study, we identified that recombinant GRA6Nt protein (rGRA6Nt) adjuvant promptly and selectively activates mRNA expression for IFN-γ and IL-18, but not IL-12 or IL-15, in innate immune cells of mice. Notably, an adjuvant that selectively activates innate production of IFN-γ and IL-18 has not been reported before. In addition, we discovered that immunizations of mice with nonreplicable (treated with mitomycin C [MMC} or irradiated) MC38 CRC cells plus rGRA6Nt adjuvant can induce a rejection of the growth of the MC38 CRC tumors after its challenge implantation of large numbers (1 or 0.5 × 10^6^ cells) of the cancer cells in more than one fifth of the immunized mice.

## Materials and Methods

### MICE

Female C57BL/6 (B6) mice and B6-background SCID mice deficient in both T and B cells (6–7 week old) were obtained from the Jackson Laboratory (Bar Harbor, ME). The studies were performed in accordance with an approved protocol (protocol #2021–3959) from the Institutional Animal Care and Use Committee.

### STIMULATION OF INNATE IMMUNE CELLS WITH rGRA6Nt *IN VIVO*

SCID mice (9–10 week old) were injected intraperitoneally with 40 μg of rGRA6Nt in 0.2 ml of PBS. At 2, 4, and 6 days after the injection, their peritoneal exudate cells (PECs) were harvested after an injection of 5 ml of PBS in their peritoneal cavity. As a control, the PECs were also harvested from one group of SCID mice before the injection of rGRA6Nt.

### PURIFICATION OF RNA FROM THE INNATE IMMUNE CELLS AND RT-PCR FOR MEASURING mRNA LEVELS FOR IFN-γ, IL-12, IL-15, AND IL-18

The PECs from the SCID mice were washed once in PBS by centrifuging them at 1,200 rpm (250 × g) for 10 minutes, followed by washing once in RMPI1640 medium (MilliporeSigma, Burlington, MA) in the same manner. After the centrifugation, the PECs in the pellets were applied for RNA purification using RNA STAT-60 (Tel-test, Friendswood, TX), followed by treatment with DNase I (Invitrogen, Waltham, MA) to remove genomic DNA contamination as described previously^21, 22^. cDNA was synthesized from 1 μg of the DNase I-treated RNA from the PEC from each mouse. Quantitative PCR with the cDNA for β-actin (a house-keeping control molecule), IFN-γ, IL-12α, IL-15, and IL-18 were performed in triplicated wells using ready-made primers and probes from Applied Biosystems (Waltham, MA)^22^.

### IMMUNIZATION OF B6 MICE WITH NONREPLICABLE MC38 CRC CELLS WITH rGRA6Nt ADJUVANT

MC38 CRC cell line (Kerafast, Boston, MA) were maintained by following the manufacture instruction. MC38 CRC cells harvested from the cultures were treated with MMC (MilliporeSigma) or irradiated to make them nonreplicable for immunizations. For MMC treatment, MC38 cells were suspended in the culture medium at 5 × 10^6^ cells/ml and incubated with MMC (100 μg/ml) for 1 hr. For the treatment with irradiation, MC38 cells suspended in HBSS with 2% fetal bovine serum at 1 × 10^6^ cells/ml were irradiated with 30 Gy by X-ray with PXI XRAD 225XL with the aluminium filter (Precision X-ray, Madison, CT) at an approximate rate of 2.2 Gy/minute. After the treatment with MMC or irradiation, MC38 CRC cells were washed and suspended in Dulbecco’s modified PBS (DPBS) (Gibco). B6 mice were immunized intraperitoneally with 1 × 10^6^ of the MMC-treated or irradiated MC38 CRC cells with 40 μg of rGRA6Nt adjuvant twice with a four-week interval. As a negative control, mice were treated with DPBS. In some experiments, a group of mice were immunized with nonreplicable MC38 CRC cells alone without any adjuvant as an additional control.

### CHALLENGE IMPLANTATION OF MC38 CRC

Two weeks after the 2nd immunization with MMC-treated or irradiated MC38 CRC cells, mice were injected subcutaneously with 1 × 10^6^ or 0.5 × 10^6^ MC38 cells in 0.1 ml of DPBS. Sizes of the tumors were measured using digital caliper every 3 or 4 days starring at Days 5 or 6 after the implantation. When the diameter of the tumor reaches 15 mm, mice were euthanized for humane reason and recorded for the time of death of those mice.

### STATISTICAL ANALYSES

Levels of significance in differences between experimental groups were determined by Student’s *t* or Mann-Whitney *U* test using GraphPad Prism software 9.0. Log-rank (Mantel-Cox) test (GraphPad Prism) was used for determining levels of significance in differences in the survival curves of mice between experimental groups. Levels of significance in numbers of mice that rejected the growth of the implanted MC38 CRC tumors were determined using Fisher Exact test (GraphPad Prism). Differences that had *p* values <0.05 were considered significant.

## Results

### rGRA6Nt OF *T. GONDII* SELECTIVELY ACTIVATES mRNA EXPRESSION FOR IFN-γ AND IL-18 IN INNATE IMMUNE CELLS

**Figure 1.**
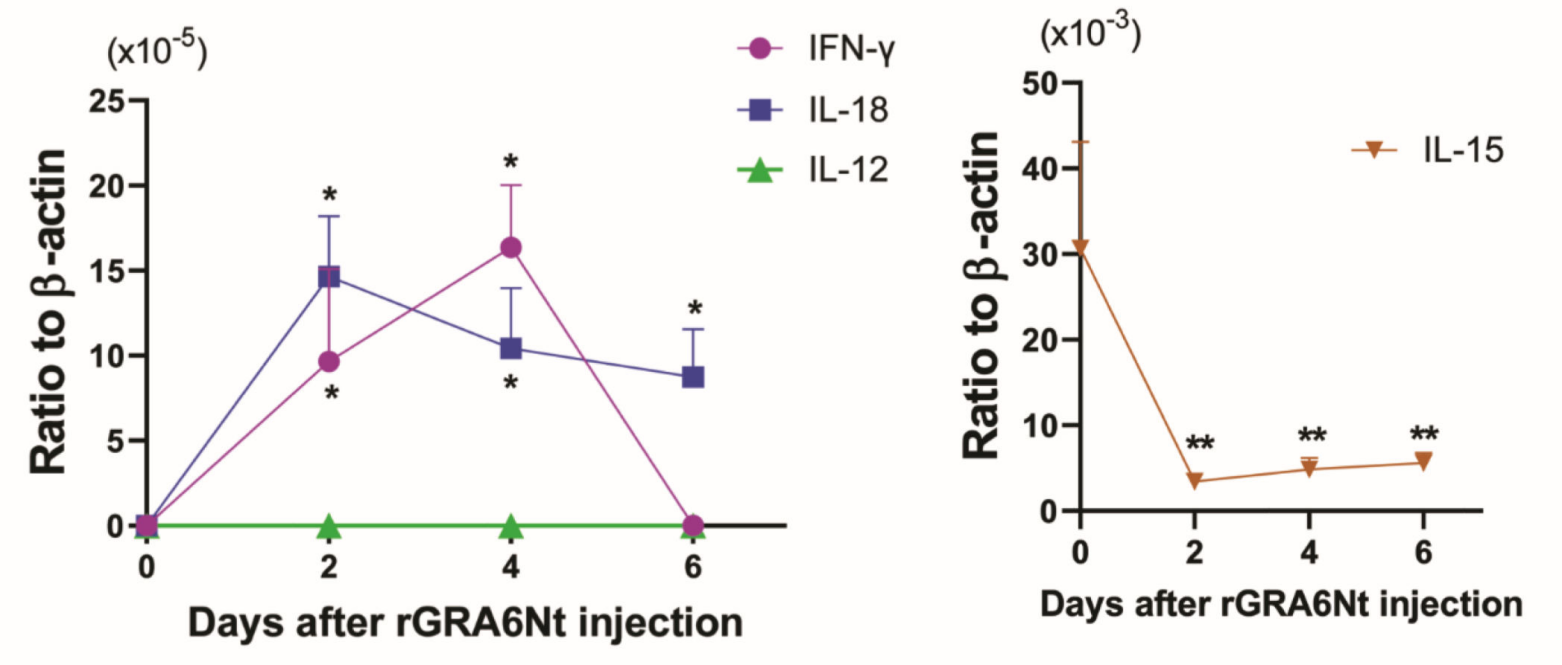
Selective upregulation of mRNA expression for IFN-γ and IL-18, but not for IL-12 and IL-15, by rGRA6Nt protein adjuvant in innate immune cells. SCID mice deficient in both T and B cells were injected intraperitoneally with 40 μg of rGRA6Nt protein adjuvant, and their PECs were harvested at Days 2, 4, and 6 after the injection. mRNA levels for IFN-γ, IL-12, IL-15, and IL-18 were measured by real time RT-PCR (n=4 at each time points). *P<0.05 and *P*<0.01 when compared to Day 0.

IFN-γ is critical for activating CD8^+^ cytotoxic T cells^17,23^. IL-12, IL-15, and IL-18 also play important roles in facilitating an activation of CD8^+^ T cells^18–20^. To determine whether rGRA6Nt activates expression of any of these cytokines in innate immune cells, B6-background SCID mice deficient in both T and B cells were injected intraperitoneally with 40 μg of rGRA6Nt, and 2, 4, and 6 days later, their peritoneal innate immune cells (peritoneal exudate cells [PECs]) were obtained for measuring their mRNA expression for IFN-γ, IL-12, IL-15, and IL-18. The PECs from unstimulated SCID mice were used as a control. Whereas mRNA for each of IFN-γ, IL-12, and IL-18 were undetectable in the control PECs (Figure 1), markedly increased levels of IFN-γ mRNA were detected in the PECs at Days 2 and 4 after rGRA6Nt injection (*P*<0.05, Figure 1). In addition, highly increased mRNA expressions for IL-18 were detected at Days 2, 4, and 6 after the rGRA6Nt injection (*P*<0.05, Figure 1). In contrast, mRNA levels for IL-12 remained undetectable at all of the three times points after the rGRA6Nt injection (Figure 1). IL-15 mRNA levels rather decreased after the rGRA6Nt injection (Figure 1). These results indicate that rGRA6Nt promptly and selectively activates expressions of mRNA for IFN-γ and IL-18 in the innate immune cells.

### IMMUNIZATIONS WITH NONREPLICABLE MC38 CRC CELLS PLUS rGRA6Nt PROTEIN ADJUVANT INDUCE A REJECTION OF THE GROWTH OF IMPLANTED TUMORS OF THE IDENTICAL CANCER CELLS IN A SIGNIFICANT PORTION OF MICE AFTER ITS CHALLENGE IMPLANTATION

Our recent study demonstrated that immunizations of immunocompetent wild-type B6 mice with nonreplicable (MMC-treated or irradiated) MC38 CRC cells plus rGRA6Nt adjuvant activate CD8^+^ cytotoxic T cells against the CRC cells and inhibit the growth of the tumors of the identical cancer cells after its challenge implantation of 1 × 10^6^ cells^16^. In the present study, we focused on whether the immunizations with rGRA6Nt adjuvant can induce a rejection of the growth of the implanted tumor cells using two different doses (1 × 10^6^ and 0.5 × 10^6^ cells) of the challenge implantation of the cancer cells.

**Figure 2.**
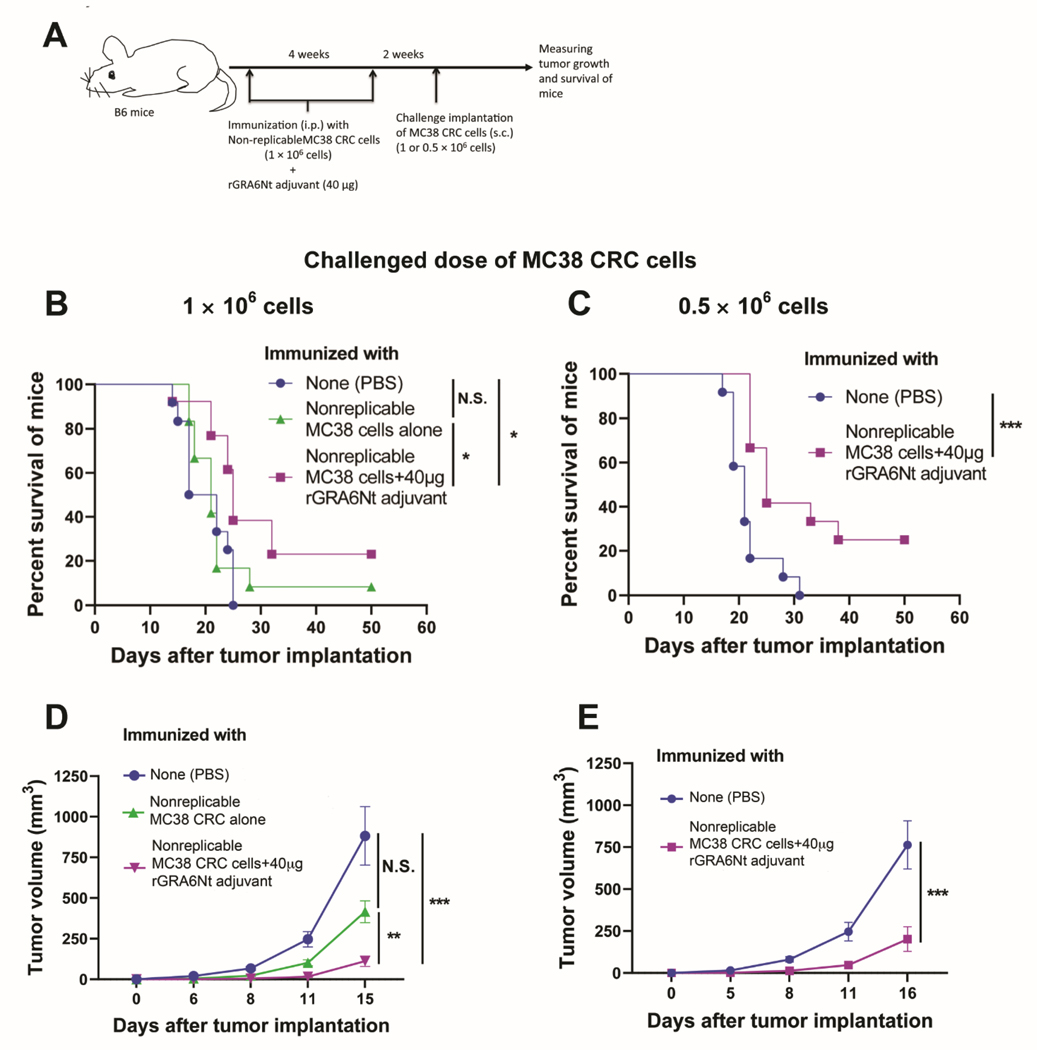
Survival rates of mice with and without immunization with nonreplicable MC38 CRC cells plus rGRA6Nt protein adjuvant after a challenge implantation of replication-capable tumor cells of the identical CRC. (**A**) a schematic figure of the experimental procedure. (**B**) The survival curves of mice and (**D**) tumor growth after the challenge implantation of 1 × 10^6^ cells of MC38 CRC cells (n=12 or 15 in each group, combined from three independent replicates). (**C**) The survival curves of mice and (**E**) tumor growth after the challenge implantation of 0.5 × 10^6^ cells of MC38 CRC cells (n=12 in each group, combined from two independent replicates). *P<0.05 and ***P<0.001.

The experimental procedures are summarized in Figure 2A. B6 mice were immunized intraperitoneally with nonreplicable (MMC-treated or irradiated) MC38 CRC cells (1 × 10^6^ cells) plus rGRA6Nt adjuvant (40 μg) twice with a 4-week interval. Additional groups of mice were immunized with phosphate-buffered saline (PBS) or nonreplicable MC38 CRC cells alone without any adjuvant as controls. Two weeks after the 2^nd^ immunization, mice were challenged with a subcutaneous injection of replication-capable MC38 CRC cells (1 × 10^6^ cells). The growth of the implanted tumors was measured by their sizes every 3 or 4 days starring at Days 5 or 6 after the implantation. When the diameter of the tumor reaches 15 mm, mice were euthanized for humane reason and recorded for the time of death of those mice.

Mice immunized with nonreplicable MC38 cells in combination with rGRA6Nt adjuvant markedly inhibited the growth of implanted tumors when compared the control mice immunized with PBS (*P*<0.001) and those immunized with nonreplicable CRC alone without any adjuvant (*P*<0.01) (Figure 2D). In addition, the immunized mice had significantly prolonged survival after the challenge implantation of the CRC cells when compared to the control mice with PBS injection as well as those immunized with nonreplicable MC38 cells without any adjuvant (*P*<0.05 for both control groups, Figure 2B). Survival time of mice immunized with nonreplicable tumor cells with no adjuvant did not differ from that of the PBS-injected control mice (Figure 2B). Notably, 20% (3/15) of the mice immunized with nonreplicable MC38 CRC cells plus rGRA6Nt adjuvant rejected the growth of the implanted tumor cells after a challenge implantation, whereas none of unimmunized control mice (0/15) was able to reject the growth of the tumors (Figure 2B).

When mice received a challenge implantation of a smaller dose (0.5 × 10^6^ cells) of MC38 CRC cells, mice immunized with nonreplicable (MMC-treated) MC38 CRC cells plus rGRA6Nt adjuvant strongly inhibited the growth of the tumors (*P*<0.001, Figure 2E), and the immunized mice survived markedly longer than the control mice with PBS injection (*P*<0.001, Figure 2C). Importantly, 25% (3/12) of the immunized mice rejected the growth of the implanted tumors (Figure 2C). This is a clear contrast to the unimmunized control group injected with only PBS, in which all mice (12/12) were unable to reject the growth of the implanted tumors (Figures 2C).

When the results from the challenge implantation of the two different doses of MC38 CRC tumor cells are combined, the rejection rate of the implanted tumors in the mice immunized with nonreplicable MC38 CRC cells plus rGRA6Nt (6/27, 22.2%) was significantly higher than that (0/27) of the unimmunized control mice (*P*<0.05, Table 1). These results indicate that the immunizations with nonreplicable (treated with MMC or irradiated) MC38 CRC cells in combination with rGRA6Nt protein adjuvant not only suppress the growth of the identical tumors after its challenge implantation but also successfully induce a rejection of the growth of the implanted tumors in more than one fifth of the mice.

## Discussion

Nucleotide- or deoxynucleotide-based adjuvants (unmethylated CpG deoxynucleotides [CpG ODN]^24–26^, a TLR9 agonist, and polyincosinic-polycytidylic acid [Poly I:C]^27, 28^, a TLR3 agonist ) are currently used for cancer immunotherapy. A major activity of CpG ODN and Poly I:C is an activation of type I IFN (IFN-α and IFN-β) and NFκB-mediated proinflammatory cytokines such as TNF-α, IL-1β, IL-6, and IL-12^29-32^. In contrast to these nucleotide- or deoxynucleotide-based adjuvants, rGRA6Nt is a protein molecule and unlikely to bind to either TLR3 o TLR9. Notably, the present study uncovered that rGRA6Nt selectively activates mRNA expression for IFN-γ and IL-18, but not for IL-12 and IL-15, in innate immune cells. To our knowledge, an adjuvant that selectively activates IFN-γ and IL-18 expression in innate immune cells has not been reported before.

IFN-γ and IL-18 play important roles in activating CD8^+^ cytotoxic T cells^17,20,23,33^. IFN-γ is also a potent activator of antigen-presenting cells such as dendritic cells to upregulate their expression of the major histocompatibility complex (MHC) class I and II molecules that present target antigens to T cells^34,35^. IL-18 also induces IFN-γ production by T cells^33,36,37^. Consistently, our recent study revealed that immunizations with nonreplicable MC38 CRC cells plus rGRA6Nt adjuvant potently activate not only CD8^+^ cytotoxic T cells but also IFN-γ production of both CD4^+^ and CD8^+^ T cells specifically to the tumor cells^16^. Notably, the present study further revealed that the immunizations with the nonreplicable MC38 CRC cells plus rGRA6Nt adjuvant induce a rejection of the growth of the implanted tumors in more than one fifth of the immunized mice, while none of non-immunized control mice rejected the growth of the implanted tumor. Therefore, rGRA6Nt is a novel protein adjuvant that has an unique activity to selectively activate production of both IFN-γ and IL-18 by innate immune cells to potently activate cancer-specific CD8^+^ cytotoxic T cells and IFN-γ production by both CD4^+^ and CD8^+^ T cells. Importantly, the immunization using this novel adjuvant is able to confer a potent protection not only to inhibit the growth of the tumors after a challenge implantation of the cancer cells identical to those used in the immunotherapy but also to induce rejection of the growth of the implanted tumors in a significant portion of mice.

The presence of tumor-infiltrating CD8^+^ T cells is an indicator of positive prognosis of cancer patients^14,15^, as mentioned earlier. In *T. gondii* infection, we recently discovered that the parasite-specific CD8^+^ cytotoxic T cells are capable of penetrating into the tissue cysts (the chronic stage form) of the parasite, which can grow into the size of more than 100 μm in diameter, to induce their destruction and elimination^38^. *T. gondii* secretes GRA6 into infected cells^39^. Therefore, when the tachyzoites (the acute stage form) of the parasite proliferates within host cells and destroy those cells during the acute stage of the infection, GRA6 present within the infected cells will be released into the tissues and most likely functions as an adjuvant to activate the CD8^+^ cytotoxic T cells capable of penetrating into the tissue cysts. Therefore, there is an intriguing possibility that rGRA6Nt functions as an adjuvant to activate CD8^+^ cytotoxic T cells capable of penetrating into solid tumors. Notably, our recent study identified that the densities of CD8^+^ T cells within MC38 CRC tumors after its challenge implantation are two-fold greater in the tumors grown in mice immunized with nonreplicable MC38 CRC cells plus rGRA6Nt adjuvant than those grown in unimmunized mice.

Although CAR T cell therapy^1^ and immune checkpoint blockage^5–8^ made a notable progress in cancer therapy, each of these approaches still have significant limitations due to difficulties in applying CAR T cell therapy to various cancer types and disadvantages due to potential severe toxic side effects of the immune checkpoint blockage because of the antigen-nonspecificity of this treatment as mentioned earlier. Therefore, it is crucial to develop a new immunotherapy that is capable of activating the protective CD8^+^ cytotoxic T cells specifically against cancer of each individual patient. In the setting of surgically resectable CRC in cancer patients, recurrence rate of curative surgically resected CRC is around 30–35%^11,12^ as mentioned earlier. Therefore, applying immunizations with nonreplicable (MMC-treated or irradiated) cancer cells of surgically-resected their own CRC in combination with an effective adjuvant to potently activate the cancer-specific CD8^+^ cytotoxic T cells would be a valuable approach to activate the protective immunity specifically against their own cancer cells and prevent recurrence of their CRC. In this regard, the present study revealed that the use of rGRA6Nt protein adjuvant in the immunization with MC38 CRC cells induces a rejection of the growth of tumors of the identical CRC cells in more than one fifth (22.2%) of the immunized mice after its challenge implantation of 1 × 10^6^ or 0.5 × 10^6^ of the cancer cells. In clinical setting, recurrence of surgically resected CRC tumors in the cancer patients would most likely start with very low numbers of the tumor cells, much lower than 1 × 10^6^ or 0.5 × 10^6^ cells used in the present study. Therefore, immunizations of CRC patients with nonreplicable cancer cells of their own surgically resected tumors with the unique rGRA6Nt protein adjuvant have excellent potential of providing a valuable pathway to prevent recurrence of the tumors in those patients.

## Conclusion

The present study revealed that rGRA6Nt protein adjuvant derived from a protozoan parasite, *T. gondii*, uniquely and selectively activates mRNA expressions for IFN-γ and IL-18, but not IL-12 and IL-15, of innate immune cells in SCID mice deficient in both T and B cells. IFN-γ and IL-18 are known to assist in activating CD8^+^ cytotoxic T cells and IFN-γ production by T cells against their target antigens. CD8^+^ cytotoxic T cells and IFN-γ are both important mediators of the protective immunity against cancers. Therefore, rGRA6Nt protein adjuvant has an ideal activity for its use in cancer immunotherapy to activate the protective T cell immunity specifically against the cancer cells. Consistently, the present study also demonstrated that immunizations with nonreplicable (treated with MMC or irradiated) MC38 CRC cells plus rGRA6Nt protein adjuvant are able to induce a rejection of the growth of the implanted tumors after a challenge implantation of replication-capable tumor cells of the identical CRC in more than one fifth of the immunized mice. Therefore, there is a possibility that immunizations of CRC patients with nonreplicable cancer cells of surgically resected their own tumors in combination with rGRA6Nt protein adjuvant could provide a novel pathway to effectively prevent recurrence of the CRC in those patients.

## Figures and Tables

**Table 1.** Rejection of the growth implanted MC38 CRC cells in mice immunized with nonreplicable MC38 CRC cells plus rGRA6Nt adjuvant

Immunization*	Frequency of tumor growth rejection*

None (PBS)	0% (0/27)
Nonreplicable MC38 alone	8.3% (1/12) N.S.
Nonreplicable MC38 CRC cells plus rGRA6Nt adjuvant	22.2% (6/27) *P*<0.05

* See the Materials and Methods section for the immuniztion procedure and measuring tumor growth rejection.
